# Dynamics of intrinsic whole-brain functional connectivity in abstinent males with methamphetamine use disorder

**DOI:** 10.1016/j.dadr.2022.100065

**Published:** 2022-05-14

**Authors:** Ping Jiang, Jiayu Sun, Xiaobo Zhou, Lu Lu, Lei Li, Jiajun Xu, Xiaoqi Huang, Jing Li, Qiyong Gong

**Affiliations:** a Huaxi MR Research Center (HMRRC), Department of Radiology, West China Hospital of Sichuan University, Chengdu, China; b Research Unit of Psychoradiology, Chinese Academy of Medical Sciences, Chengdu, China; c Functional and Molecular Imaging Key Laboratory of Sichuan Province, Chengdu, China; d Department of Radiology, West China Hospital of Sichuan University, Chengdu, China; e Department of Psychosomatics, Academy of Medical Sciences & Sichuan Provincial People's Hospital, Chengdu, Sichuan, China; f Mental Health Center, West China Hospital of Sichuan University, Chengdu, China

**Keywords:** Dynamic functional connectivity, Methamphetamine, Resting-state fMRI, Neural networks, Independent component analysis

## Abstract

**Background:**

The global prevalence of methamphetamine use disorder (MUD) and the associated economic burden are increasing, but effective pharmacological treatment is lacking. Therefore, understanding the neurological mechanisms underlying MUD is essential to develop clinical strategies and improve patient care. Individuals with MUD can show static brain network abnormalities during the resting state, but their alterations in dynamic functional network connectivity (dFNC) are unclear.

**Methods:**

In this study, we obtained resting-state functional magnetic resonance imaging from 42 males with MUD and 41 healthy controls. Sliding-window and spatial independent component analyses with a *k*-means clustering algorithm were used to assess the recurring functional connectivity states. The temporal properties of the dFNC, including fraction and dwelling time of each state and the number of transitions between different states, were compared between the two groups. In addition, the relationships between the temporal properties of the dFNC and clinical characteristics of the MUDs, including their anxiety and depressive symptoms, were further explored.

**Results:**

While the two groups shared many similarities in their dFNC, the occurrence of a highly integrated functional network state and a state featuring balanced integration and segregation in the MUDs significantly correlated with the total drug usage (Spearman's rho = 0.47, *P* = 0.002) and duration of abstinence (Spearman's rho = 0.38, *P* = 0.013), respectively.

**Conclusions:**

The observed results in our study demonstrate that methamphetamines can affect dFNC, which may reflect the drug's influence on cognitive abilities. Our study justifies further studies into the effects of MUD on dynamic neural mechanisms.

## Highlights

• This study observed the temporal properties of dynamic functional network connectivity (dFNC) in abstinent males with methamphetamine use disorder.
• The choice of a cohort with single sex and only METH use was important to eliminate the confounding effects of multiple substances use and sex differences.
• Drug use history is significantly associated with the occurrence of the functional network states, suggesting the neurotoxic effect of METH overuse on the dFNC properties.

## 1 Introduction

The use of methamphetamine (METH), an amphetamine-type stimulant, has been increasing rapidly worldwide (UNODC, 2019). In fact, METH has become the primary drug of abuse in China, according to the 2018 National Survey on Drug Use in China. The high prevalence of METH use, together with the high recurrence rate among persons with methamphetamine use disorder (MUD), constitutes a substantial public health burden in China and worldwide.

Existing medication-based interventions cannot effectively treat MUD (Gouzoulis-Mayfrank et al., 2017; London, 2016; UNODC, 2019). Thus, understanding the neurological mechanisms underlying MUD is critical for developing clinical strategies that reduce recurrence. Such an understanding can come from functional magnetic resonance imaging (fMRI) (Gong et al. 2021), which can detect alterations in connectivity among cortical areas that function together as brain networks (Fox et al., 2005; Smith et al., 2009). Resting-state fMRI has been applied extensively to patients with psychiatric disorders (Haber et al., 2020; Keshavan et al., 2020; Canario et al. 2021), and studies of patients with MUD have identified dysfunction in the default mode network (Ipser et al., 2018) and motive circuit (Mansoory et al., 2020), as well as altered topological graph properties (Siyah Mansoory et al., 2017). Our own resting-state fMRI study showed disrupted functional connectivity in salience and cerebellar networks in individuals with MUD (Jiang et al., 2021). We further showed in that work that static functional connectivity of the rostral anterior cingulate cortex in the ventral medial network mediates the association between duration of abstinence and the severity of affective symptoms, i.e., anxiety and depression, in the MUDs.

While these resting-state studies have provided insights into METH effects on static brain networks, they cannot detect dynamic changes in functional connectivity (Abrol et al., 2017; Allen et al., 2014; Preti et al., 2017). Indeed, many neurological and psychiatric disorders involve alterations in so-called dynamic functional network connectivity (dFNC). These disorders include Parkinson's disease (Fiorenzato et al., 2019; Kim et al., 2017) and major depressive disorder (Long et al., 2020). One previous study found abnormal time-domain differences in resting-state fMRI in individuals with MUD during abstinence (Dong et al., 2021); however, the detailed alteration of dFNC and its relationship to the clinical characteristics of MUD are unclear. Therefore, in the present study, we used resting-state fMRI and a sliding-window analysis to compare dFNC between males with MUD during abstinence and age- and sex-matched healthy controls. Moreover, we explored the relationships between the temporal properties of dynamic connectivity and the clinical characteristics of males with MUD, including their affective symptoms.

## 2 Experimental procedures

### 2.1 Participants

Forty-eight male Han Chinese with MUD (mean age ± SD = 29.45 ± 7.67 years) were consecutively recruited from Ziyang Compulsory Isolation and Rehabilitation Center of Sichuan Province, China, between January and December 2015. Subjects recruited in our study were required to be older than 16 years old, fulfill the criteria for METH abuse based on the Structured Diagnostic Interview for DSM-IV Disorders, and be able to complete the measurements. We examined only males with MUD without a history of using other illicit substances to eliminate confounding effects of sex and the use of multiple substances.

Exclusion criteria included (1) a history of mental disorder(s) before METH use or any psychoactive substance use other than METH or nicotine; (2) current severe medical diseases requiring frequent medical visits or inpatient treatment; (3) current use of medications that affect hemodynamics, e.g., insulin or thyroid medication; (4) history of head injuries involving loss of consciousness or other neurological disorders, such as stroke or Parkinson's disease; and (5) contraindications to MRI, such as metal implant, use of a cardiac pacemaker or claustrophobia.

Forty-eight healthy Han Chinese controls (mean age ± SD = 28.10 ± 9.70 years), matched to the patients by age and sex, were recruited from the imaging database of Huaxi Magnetic Resonance Research Center and through posters and flyers distributed at West China Hospital of Sichuan University. Healthy participants were recruited if they did not present any drug use history other than nicotine and if they showed no indications of neurologic or psychiatric illness based on medical records or T1-weighted MRI.

This study was approved by the Ethics Committee of the West China Hospital of Sichuan University in Sichuan, China. Written informed consent was obtained from all participants after a full explanation of the study procedure.

### 2.2 Clinical assessment

Detailed interviews including questions on socio-demographics, drug use history and anxiety and depressive symptoms by the Hamilton Anxiety Rating Scale (HAM-A) and Hamilton Depression Rating Scale (HAM-D) were conducted with patients by experienced psychiatrists (X.Z. and L.N.). The HAM-A is a 14-item questionnaire with a total score ranging from 0 to 56 (Hamilton, 1959; Vaccarino et al., 2008). The HAM-D is a 17-item questionnaire with a total score ranging from 0 to 52 (Hamilton, 1960). Higher total scores of the scales indicate more severe symptoms.

### 2.3 MR imaging acquisition

MR images were acquired on a 3.0-T Tim Trio scanner (Siemens Healthineers, Erlangen, Germany) with a 12-channel head coil. A three-dimensional T1-weighted image was acquired using a spoiled gradient recalled echo sequence with the following parameters: repetition time (TR) = 1900 ms, echo time (TE) = 2.26 ms, flip angle = 9°, voxel size = 1 × 1 × 1 mm^3^, matrix resolution = 256 × 256, field of view = 256 × 256 mm^2^, slice thickness = 1 mm, and number of slices = 176. Resting-state fMRI data were acquired using a gradient-echo echo-planar imaging sequence with the following parameters: TR = 2000 ms, TE = 30 ms, flip angle = 90°, voxel size = 3.75 × 3.75 × 5 mm^3^, matrix size = 64 × 64, field of view = 240 × 240 mm^2^, slice thickness = 5 mm without intersection gaps, and axial slices = 30. The functional imaging session contained 205 image volumes, resulting in a total imaging time of 6 min 50 s. Participants were instructed to keep their eyes closed and not to concentrate on anything in particular during the acquisition. Earplugs were used to attenuate scanning noise, and foam pads were used to minimize head motion. An experienced neuroradiologist (J. S.) evaluated scans for clinical abnormalities and verified the structural image quality.

### 2.4 Preprocessing of resting-state fMRI data

Individual resting-state fMRI data were preprocessed using the FSL analysis package (FMRIB Software Library, Oxford, UK). Preprocessing consisted of exclusion of the first 5 volumes, brain extraction, slice timing correction, motion correction, high-pass temporal filtering equivalent to 100 s (0.01 Hz) and spatial smoothing using a Gaussian kernel with a full width at half-maximum of 5 mm. Functional MRI data were registered to the individual's structural image and the MNI152 standard space template with 2 mm spatial resolution using the boundary-based registration (BBR) method as implemented in the FMRIB Linear Image Registration Tool (FLIRT) (Jenkinson et al., 2002; Jenkinson and Smith, 2001). Variance in voxel time series was normalized across space to minimize bias during subsequent variance-based data reduction steps (Allen et al., 2014).

### 2.5 Image quality and motion control

As dFNC analyses are sensitive to head motion, we applied stringent criteria to minimize such confounding. First, mean framewise displacement (FD) values across translational and rotational directions were calculated between successive images based on six rigid-motion parameters obtained during realignment steps of each subject (Power et al., 2012). Participants with a mean FD value > 0.5 mm or maximum FD > 2 mm were excluded from the analysis. Based on these criteria, we excluded six MUDs and seven healthy controls from subsequent analyses.

Second, the FMRIB independent component analysis (ICA)-based Xnoiseifier—FIX (version 1.061 beta; Griffanti et al., 2014; Salimi-Khorshidi et al., 2014)—was applied to each individual's resting-state fMRI data to control for head motion and other nuisance noise, such as due to respiration or heartbeat, and to produce clean datasets for the subsequent analyses. The level of head motion-related noise was significantly reduced, as shown by the comparison between the mean FD value before and after FIX denoising within each group (*P* < 0.0001), but there were no significant intergroup differences in mean FD values either before or after denoising (both *P* > 0.05).

### 2.6 Group independent component analysis

After data preprocessing, resting-state fMRI data of 42 patients and 41 healthy controls were analyzed using a group-level spatial ICA as implemented in the GIFT toolbox (version 4.0b; http://mialab.mrn.org/software/gift/). Two data reduction steps were performed in the principal component analysis. First, subject-specific data were reduced to 150 components, and the subject-reduced data were concatenated over time. Second, at the group level, data were reduced to 70 group independent components using the expectation-maximization algorithm (Roweis, 1998). The FastICA algorithm in ICASSO with 10 repetitions was used to ensure reliability and stability of the components (Hyvarinen, 1999). The resulting components were clustered to estimate their reliability, and components with average intra-cluster similarity values > 0.70 were selected. Subject-specific time courses and spatial maps were generated using the back-reconstruction approach GICA (Calhoun et al., 2001).

Among the 70 independent components, we identified intrinsic networks based on visual inspection and spatial correlation with other templates from previous work (Shirer et al., 2012; Smith et al., 2009). This procedure resulted in 39 meaningful components, which we sorted into seven functional networks: subcortical, auditory, sensorimotor, visual, default mode, executive control, and cerebellar networks.

To remove residual noise, the time courses of the 39 independent components were detrended, despiked using AFNI's 3dDespike algorithm, and filtered using a fifth-order Butterworth low-pass filter with a cutoff of 0.15 Hz.

### 2.7 Analysis of temporal properties of dFNC

dFNC between different brain networks was estimated by the sliding window approach as implemented in the GIFT toolbox (https://trendscenter.org/software/gift/). The sliding window approach was used to explore time-varying changes in functional connectivity within the 39 independent components. Resting-state fMRI time series were segmented into a 22-TR window with a size of 44 s (width = 20 repetition times), which was convolved with sigma = 3 TRs of Gaussian. The window was slid stepwise by 1 TR along the 200-TR length scan, resulting in 39 × 39 consecutive windows across the entire scan. Using the time series of all possible 741 independent component pairs within each window, the resulting 179 × 741 pairwise covariance matrix was calculated using L1 regularization. Finally, values in the resulting functional connectivity matrices were converted to z scores using Fisher's z transformation for further statistical analyses.

### 2.8 Clustering analysis

To assess recurring functional connectivity patterns (i.e., connectivity states) in terms of the frequency and structure of those states, we applied a *k*-means clustering algorithm on windowed functional connectivity matrices. The L1 distance (i.e., Manhattan distance) function was used to estimate the similarity between window functional connectivity matrices. This clustering approach was applied twice to all subjects’ connectivity matrices: first, to determine the optimal number of clusters *k* (referred to as “states”); second, to construct the final *k* connectivity states. The elbow criterion of the cluster validity index was used to determine the number of clusters *k* (Allen et al., 2014). Notably, this analysis does not guarantee that all participants visit every connectivity state; for example, some might spend time in only one or two states, even though three or more states exist.

### 2.9 Statistical analysis of dynamic connectivity measures

The following dynamic connectivity measures were used to compare the individuals with MUD and healthy controls using *t* tests: (1) fraction time (FT), the percentage of total time a subject spent in a given state; (2) dwelling time (DT), the time a subject spent in one connectivity state before switching to another state; and (3) numbers of transitions from one state to another. The level of significance was set at *P* < 0.05 after correction for the false discovery rate (FDR).

### 2.10 Clinical data analysis

Statistical analyses of clinical data were performed using SPSS Statistics 23 (IBM, Chicago, IL, USA). Spearman correlation analysis was performed between dFNC measures on the one hand (FT, DT, and numbers of transitions) and history of METH use and affective measurements (i.e., HAM-A and HAM-D scores) on the other hand. Age and years of education served as covariates in the correlation analysis. Possible outliers were identified based on Cook's distance (Cook 1977), and these were excluded from the correlation analysis. Statistical significance for correlation analyses was set at a threshold of *P* ≤ 0.05/4 = 0.013 with Bonferroni correction for multiple comparisons.

## 3 Results

### 3.1 Demographic and clinical characteristics of the study groups

Table 1 lists the demographic and clinical characteristics of the 42 males with MUD and 41 healthy controls included in the study. The two groups were similar in age (*P* > 0.10) but differed significantly in years of education (*P <* 0.001). The average abstinence duration for the males with MUD was 114.69 days (SD = 114.04 days).

**Table 1:** Demographic and clinical data on MUDs and healthy controls

Characteristic	MUDs (N = 42)	Healthy controls(N = 41)
***Demographics***		
Age (years)	29.45±7.67	28.10±9.70
Education (years)	8.19±3.93	11.95±3.03⁎⁎⁎
***Affective symptoms***		
HAM-A scores	4.07±5.25	-
HAM-D scores	5.55±5.30	-
***METH use***		
Abstinence duration (days)	114.69±114.04 (range 12-400)	-
Use duration (months)†	45.68±37.83	-
Mean dose (g/time)	0.37±0.27	-
Total amount (kg)†	0.89±1.88	-
Age at first use (years)†	25.56±8.28	-

All data are expressed as mean ± standard deviation, unless otherwise noted.

Abbreviations: HAM-A, Hamilton Anxiety Rating Scale; HAM-D, Hamilton Depression Rating Scale; METH: methamphetamine; MUD: methamphetamine use disorder; g, gram; kg, kilogram.

† N = 41.

⁎⁎⁎ *P* < 0.001

### 3.2 Intrinsic connectivity networks

**Fig. 1:**
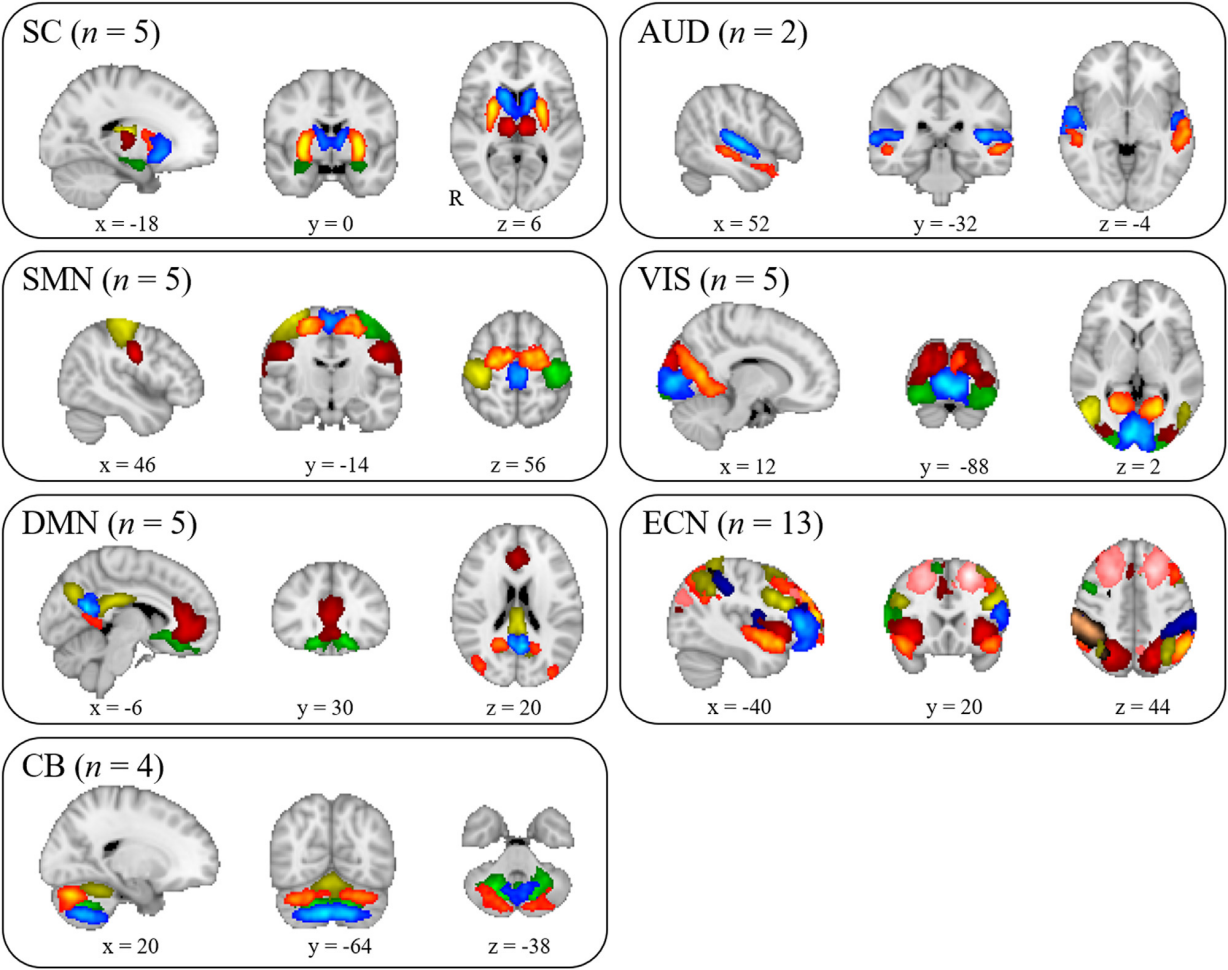
Spatial maps of 39 intrinsic connectivity networks identified by group ICA. SC, subcortical; VIS, visual; SMN, sensorimotor; AUD, auditory; DMN, default mode; ECN, executive control; and CB, cerebellar networks. *n* indicates the number of components included in the network.

Fig. 1 displays the 39 intrinsic connectivity networks identified by group ICA. Based on their anatomical and presumed functional properties, intrinsic connectivity networks were grouped into the following seven networks: subcortical, auditory, sensorimotor, visual, default mode, executive control, and cerebellar networks.

### 3.3 Clustering and analysis of dynamic functional connectivity states

**Fig. 2:**
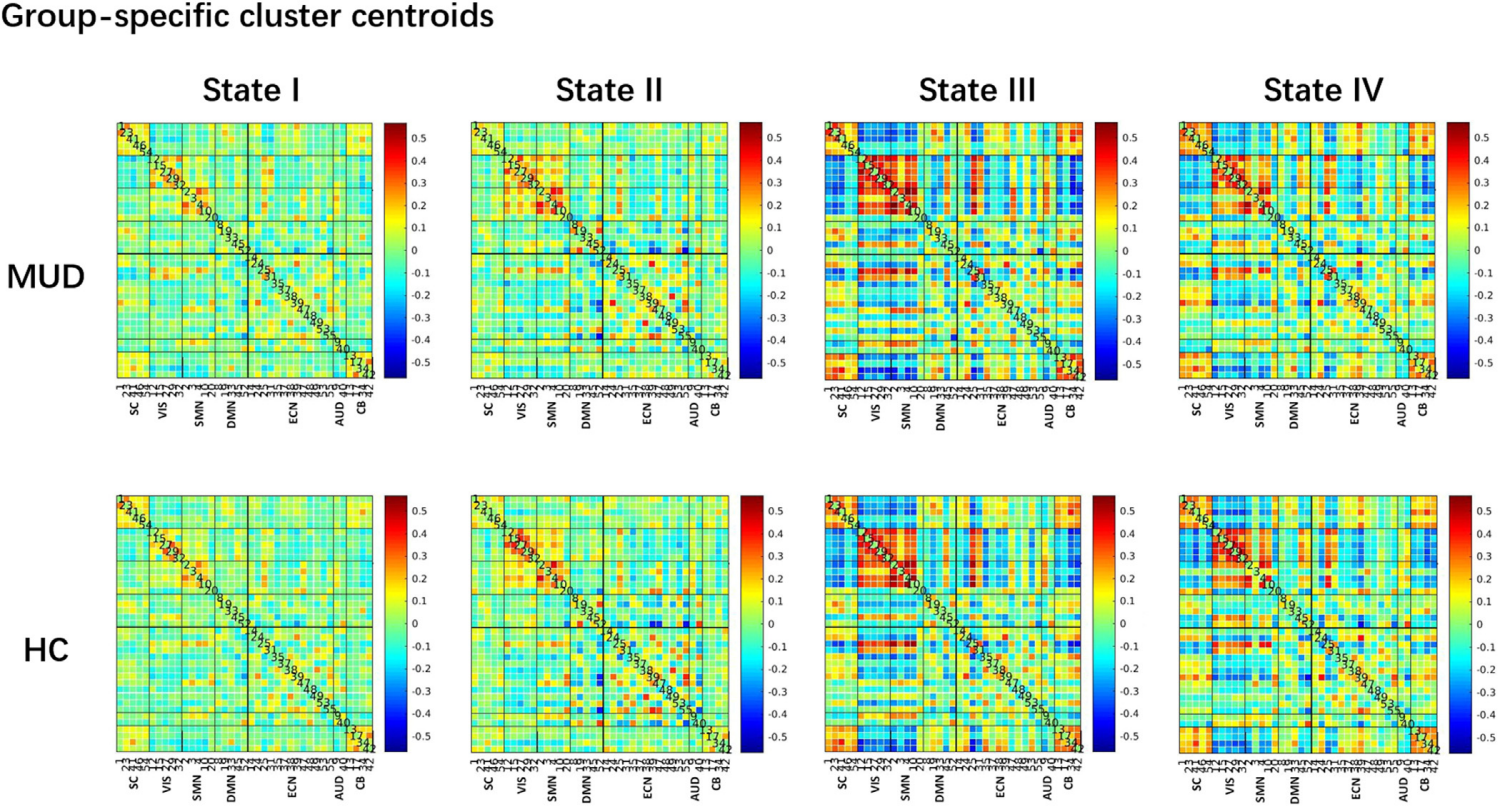
Dynamic functional connectivity states in males with methamphetamine use disorder (MUD) and healthy controls (HC), as represented as centroids of the dynamic states obtained from *k*-means clustering. Intrinsic connectivity networks are labeled subcortical (SC), visual (VIS), sensorimotor (SMN), auditory (AUD), default mode (DMN), executive control (ECN), and cerebellar (CB) networks.

Using the *k*-means clustering method, we identified four highly constructed functional connectivity states that recurred throughout individual scans and across subjects. Fig. 2 displays group-specific cluster centroids from the *k*-means clustering analysis. Functional connectivity states were arranged in order of emergence. The FT of the four states accounted for 25.03%, 32.31%, 22.16%, and 20.50% of all windows in the males with MUD, and for 28.44%, 34.35%, 19.78%, and 17.43% of all windows in healthy controls. State I appeared to involve the most integrated functional connectivity pattern; state Ⅱ appeared more segregated, especially in the unimodal networks; and state Ⅲ showed the greatest segregation. State IV showed a segregation intermediate between states Ⅱ and Ⅲ.

### 3.4 dFNC differences between individuals with MUD and controls

**Fig. 3:**
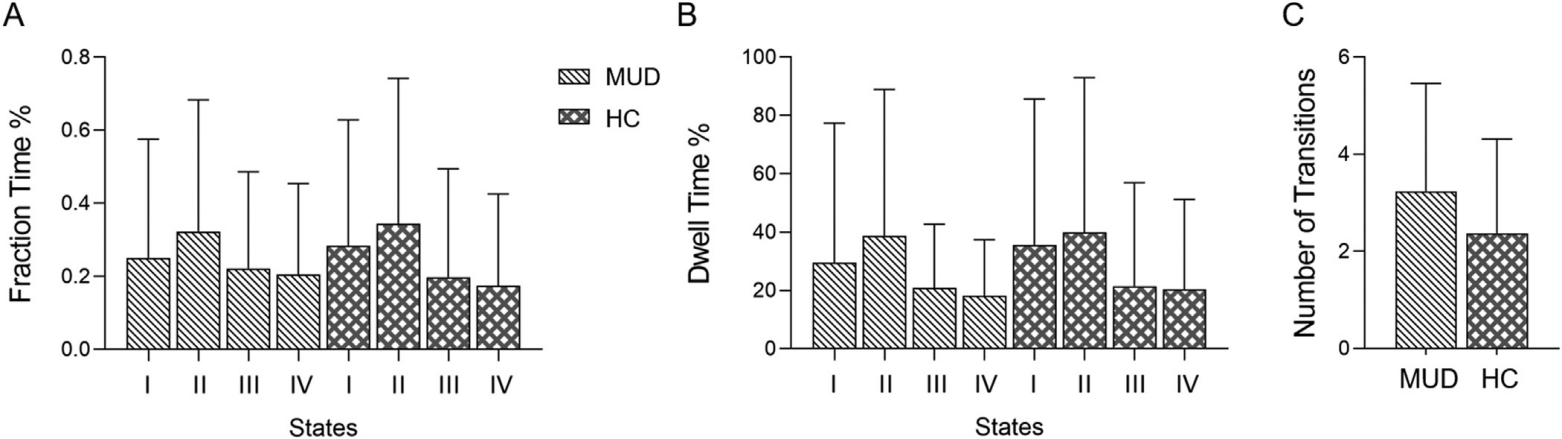
Dynamics of functional connectivity states in males with methamphetamine use disorder (MUD) and healthy controls (HCs). (A) Fraction time, defined as the percentage of total time a subject spent in a given state. (B) Mean dwell time, defined as the fraction of total time continuously spent in each state. (C) Number of transitions between states.

The dynamics of the functional connectivity states did not differ significantly between the males with MUD and controls (Fig. 3). Within each group, FT and mean DT did not differ significantly across the four states.

### 3.5 Relationships between dFNC and clinical characteristics of individuals with MUD

**Fig. 4:**
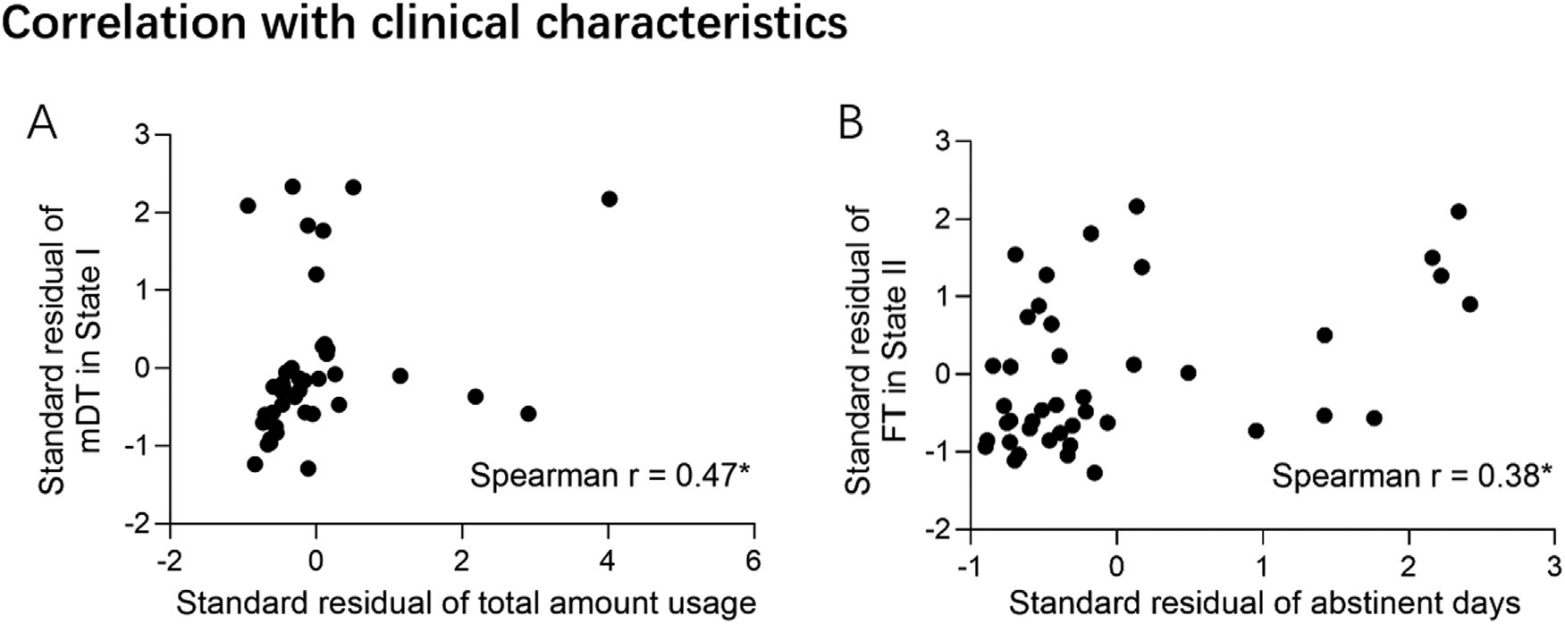
Correlation between dynamic functional network connectivity and clinical characteristics of individuals with methamphetamine use disorder. mDT, mean dwell time; FT, fraction time. * *P* ≤ 0.013

In correlation analysis in which age and years of education were treated as covariates, we found that total METH usage positively correlated with the mean DT of state Ⅰ (Spearman's rho = 0.47, *P* = 0.002, Fig. 4A), while abstinence duration positively correlated with the FT of state II (Spearman's rho = 0.38, *P* = 0.013, Fig. 4B). No outliers were detected in the correlation analysis based on Cook's distance. No significant correlations were found between dFNC features and affective measurements in the MUDs.

## 4 Discussion

Alterations in dFNC remain largely unexplored in individuals with MUD. In this study, we compared dFNC properties between males with MUD and age- and sex-matched healthy controls in terms of FT, DT, and number of transitions. We identified four distinct functional connectivity states across the whole resting state period. There was no significant group difference between the males with MUD and healthy controls in any of the dFNC properties. However, the FT and DT of certain functional network states correlated significantly with the total METH usage and duration of abstinence of the MUDs. This provides evidence that METH overuse may affect the dFNC properties of MUDs, suggesting that dFNC properties may serve as biomarkers for diagnosing MUD.

The four functional network states described here represent different integration and segregation levels of brain networks (Wang et al., 2021). These states were detected in the resting state, indicating that brain networks experience dynamic changes even without any external stimuli (Allen et al., 2014). In state Ⅰ, the functional connectivity strengths among different brain network pairs were similarly positive, representing a coactivation mode involving the whole brain as a single module. In state Ⅱ, the unimodal networks (auditory, visual, and sensorimotor) showed higher positive within-network correlations than state Ⅰ, reflecting greater segregation. State Ⅲ demonstrated the highest within-network positive functional connectivity and between-network negative functional connectivity, especially between the unimodal networks and others; thus, this state showed the lowest integration and highest segregation among the four connectivity states. The functional connectivity strengths of networks in state Ⅳ fell between those in states Ⅱ and Ⅲ, indicating intermediate network integration and segregation. Subjects spent the most time in state Ⅱ, featuring moderate network segregation and integration; this state was occupied during approximately one-third of the scanning period. This finding is consistent with recent work suggesting that healthy young brains are configured to maintain a balance between segregation and integration of brain networks in the resting state (Wang et al., 2021). However, one recent study reported a different number of functional network states (i.e., 5 states) in resting-state fMRI data of MUDs (Dong et al., 2021). The possible reasons for the discrepancy may be ascribed to the different clinical characteristics of the recruited subjects in terms of age, sex, and abstinence durations of the MUDs and various approaches for scanning parameters and data processing methods applied in the two studies.

Our failure to detect group-level dFNC differences between the males with MUD and controls may reflect that the MUDs varied in abstinence duration, during which they may have experienced different degrees of brain functional recovery (Wang et al., 2013). In addition, varied METH usage also provides different effects on brain functions (Alicata et al., 2009; Thanos et al., 2016) and thus confounds the group comparison results. Future studies should verify and extend our findings in longitudinal studies involving MUDs who used similar METH amounts and have been abstaining for shorter and longer periods.

Our correlation analyses found that among the MUDs, total drug usage positively correlated with the mean DT of state Ⅰ, while the duration of abstinence positively correlated with the FT of state Ⅱ. These observations suggest that METH overuse can influence the temporal properties of dFNC. Chronic METH administration can result in neurotoxic effects in monoaminergic neurotransmitter systems, including dopamine and serotonin, abnormalities in glucose metabolism and deficits in brain activity and functional connectivity. These aberrents accompany cognitive and motor deficits and emotional symptoms (Baicy & London, 2007; Koob & Volkow, 2016; Jiang et al., 2021). However, after protracted abstinence, individuals with MUD exhibit improved cognitive ability and decreased severity of emotional symptoms (Jiang et al., 2021; Wang et al., 2013), which may be related to significant recovery of dopamine transporter losses (Volkow et al., 2001). Previous studies investigated dopaminergic medication effects on resting-state fMRI and demonstrated that alterations in dopaminergic systems affect functional connectivity strength and small-world topology of functional brain networks (Berman et al., 2016; Esposito et al., 2013; Tahmasian et al., 2015). As the state Ⅱ is featured with relatively balanced network segregation and integration that appears to be a characteristic of the healthy brain (Wang et al., 2021), the observed positive correlations between abstinence duration and time experienced in state Ⅱ may reflect gradual recovery of dopaminergic systems from the neurotoxic effects of METH overuse after prolonged abstinence in MUDs. Conversely, greater total METH use was associated with more time spent in highly integrated state Ⅰ, perhaps reflecting the neurotoxic effects of drug abuse on the monoaminergic neurotransmitter systems. On the other hand, we found no associations between dFNC properties of MUDs and their affective symptoms. One possible explanation is that the MUDs had abstained for different periods, and they reported different severities of affective symptoms; in fact, some patients did not report any such symptoms. Larger, longitudinal studies should further explore whether dFNC correlates with METH-induced affective symptoms.

Neuroimaging studies have shown that the integration of brain networks reflects people's abilities to perform cognitive tasks, since higher brain functions rely on the ability to flexibly integrate information across specialized “communities” of brain regions (Cole et al., 2014; Jiang et al., 2018; Petersen and Sporns, 2015). Thus, a state of integration enables faster, more accurate performance on cognitive tasks (Shine et al., 2016). Previous clinical and animal studies have shown that chronic METH overuse produces cognitive dysfunction (Dean et al., 2013; Nordahl et al., 2003; Potvin et al., 2018; Sabrini et al., 2019). Therefore, the observed relationships between the patients’ clinical characteristics and the occurrence of functional connectivity states with different integration levels may indirectly reflect the influence of METH overuse on the changes in the patients’ cognitive abilities. Unfortunately, we did not measure the patients’ cognitive abilities in this study; therefore, we cannot make any conclusions from our current observation. Future studies using dFNC analysis and cognitive tasks on MUDs are needed to further confirm our assumptions.

In this study, we recruited only male MUDs to eliminate sex effects on the investigations of dFNC disturbances. Convergent evidence has indicated that males and females may be affected differently by METH use in various aspects, including emotional symptoms, cognitive abilities and brain morphometry and functions. Specifically, females showed higher rates of comorbidity with psychiatric symptoms, including depression and anxiety (Semple, Grant, & Patterson, 2004; Zweben et al., 2004), whereas male MUDs demonstrated greater deficits in memory and learning (Chang et al., 2005) and executive functions (Kim et al., 2005). On the other hand, neuroimaging studies have reported sex differences in the alterations of regional cerebral glucose metabolism (Kim et al., 2005) and gray matter volumes (Kogachi et al., 2017) in METH users. The reasons for the sex differences in the effects of METH overuse are unknown. Potential reasons could be the differential interaction of METH with sex hormones or organizational sex effects on the brain (Dluzen & Liu, 2008; Dluzen & McDermott, 2006). Future works with female MUDs and investigations on sex differences are needed to reveal the underlying biological mechanisms.

Our study presents several limitations. First, it had a cross-sectional design and thus cannot establish causal relationships between drug use and brain function. Second, our observation of a correlation between dFNC and total METH usage should be verified in further work given that the individuals with MUD in our study may have experienced different extents of recovery. Third, our analyses may have been limited by the temporal resolution and total acquisition time during fMRI. Faster fMRI acquisition with longer sampling may provide more accurate measurements of dFNC in the sliding window approach. Finally, since we obtained the rs-fMRI data with subjects eyes closed, we could not identify and exclude the subjects who fell asleep during the data acquisition, which may have confounded our final results. Therefore, rs-fMRI data obtained with subjects’ eyes open or fixing on a cross are needed to confirm our investigations in the future.

## 5 Conclusions

Our comparison of dFNC between abstinent males with MUD and healthy controls and the correlation analysis found that higher total drug use and longer abstinence correlate with more time spent in a highly integrated functional network state and a state featuring balanced integration and segregation, respectively. Our findings provide evidence that METH overuse affects the temporal characteristics of dFNC, which could serve as a diagnostic and recovery biomarker for individuals with MUD. Future work should combine the dynamic approach with cognitive testing to understand whether and how the drug's effects on dFNC lead to cognitive dysfunction.

## Role of funding source

This study was supported by the 10.13039/501100001809National Natural Science Foundation (Grant Nos. 81621003 and 82027808), 10.13039/501100012166National Key Research and Development Program of China (Grant No. 2017YFC1310401), and 10.13039/501100004829Department of Science and Technology of Sichuan Province (Grant No. 2017HH0059) of China. The founders had no further role in study design; in the collection, analysis and interpretation of data; in the writing of the report; and in the decision to submit the paper for publication.

## Contributors

Ping Jiang, Jing Li, and Qiyong Gong contributed to conception and study design. Jiayu Sun, Xiaobo Zhou, Ping Jiang, Lu Lu, Lei Li, Jiajun Xu contributed to the data acquisition. Ping Jiang contributed to the statistical analysis and drafted the manuscript. All authors contribute to interpret the results and gave final approval of the version to be published.

## Declaration of Competing Interest

The authors declare no conflicts of interest.
